# Comparative analysis of circulating metabolomic profiles identifies shared metabolic alterations across distinct multistressor military training exercises

**DOI:** 10.1152/physiolgenomics.00008.2024

**Published:** 2024-05-13

**Authors:** Michael Daniels, Lee M. Margolis, Jennifer C. Rood, Harris R. Lieberman, Stefan M. Pasiakos, J. Philip Karl

**Affiliations:** ^1^Military Nutrition Division, United States Army Research Institute of Environmental Medicine, Natick, Massachusetts, United States; ^2^Oak Ridge Institute for Science and Education, Oak Ridge, Tennessee, United States; ^3^Pennington Biomedical Research Center, Baton Rouge, Louisiana, United States; ^4^Office of Dietary Supplements, National Institutes of Health, Bethesda, Maryland, United States

**Keywords:** exerkines, ketogenesis, lipid metabolism, oxidative stress, physical activity

## Abstract

Military training provides insight into metabolic responses under unique physiological demands that can be comprehensively characterized by global metabolomic profiling to identify potential strategies for improving performance. This study identified shared changes in metabolomic profiles across three distinct military training exercises, varying in magnitude and type of stress. Blood samples collected before and after three real or simulated military training exercises were analyzed using the same untargeted metabolomic profiling platform. Exercises included a 2-wk survival training course (ST, *n* = 36), a 4-day cross-country ski march arctic training (AT, *n* = 24), and a 28-day controlled diet- and exercise-induced energy deficit (CED, *n* = 26). Log_2_-fold changes of greater than ±1 in 191, 121, and 64 metabolites were identified in the ST, AT, and CED datasets, respectively. Most metabolite changes were within the lipid (57–63%) and amino acid metabolism (18–19%) pathways and changes in 87 were shared across studies. The largest and most consistent increases in shared metabolites were found in the acylcarnitine, fatty acid, ketone, and glutathione metabolism pathways, whereas the largest decreases were in the diacylglycerol and urea cycle metabolism pathways. Multiple shared metabolites were consistently correlated with biomarkers of inflammation, tissue damage, and anabolic hormones across studies. These three studies of real and simulated military training revealed overlapping alterations in metabolomic profiles despite differences in environment and the stressors involved. Consistent changes in metabolites related to lipid metabolism, ketogenesis, and oxidative stress suggest a potential common metabolomic signature associated with inflammation, tissue damage, and suppression of anabolic signaling that may characterize the unique physiological demands of military training.

**NEW & NOTEWORTHY** The extent to which metabolomic responses are shared across diverse military training environments is unknown. Global metabolomic profiling across three distinct military training exercises identified shared metabolic responses with the largest changes observed for metabolites related to fatty acids, acylcarnitines, ketone metabolism, and oxidative stress. These changes also correlated with alterations in markers of tissue damage, inflammation, and anabolic signaling and comprise a potential common metabolomic signature underlying the unique physiological demands of military training.

## Graphical abstract

**Figure:**
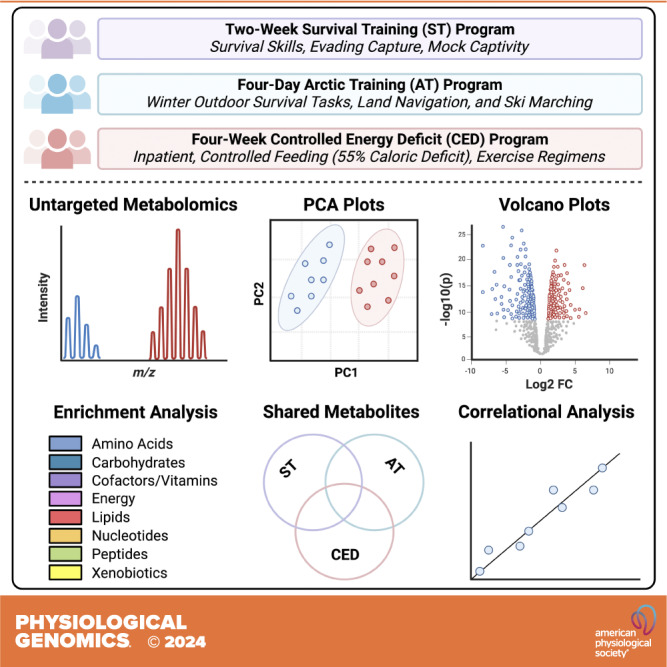

## INTRODUCTION

Military training exercises typically involve sustained physical exertion, limited sleep, energy deficit, and psychological stress (1). The training is also unique as it combines aerobic and anaerobic exercise, thus contributing to a mixed metabolic phenotype (2), and provides little opportunity for the substantive recovery often afforded to comparable populations such as athletes (3). Arduous training environments and limited recovery result in negative physiological consequences leading to decreased body mass, altered circulating hormone and substrate concentrations, and increased inflammation (4). Studying military personnel engaged in various types of training therefore provides opportunities to examine metabolic responses under unique physiological demands known to activate proinflammatory and catabolic pathways that can impair performance (5).

Incorporating high-throughput metabolomics analysis into military training environments allows for the identification of unique metabolomic signatures that can be used to monitor metabolic status during training, characterize the underlying physiology of performance decrements, and inform the development of interventions aiming to improve performance and facilitate recovery. Although relatively few studies have conducted metabolomic profiling within the context of military training, our laboratory has now done so with several real and simulated military training exercises differing in duration, source, and magnitude of physiological stressors involved (6–11). These studies have reported robust alterations in metabolomic profiles following varied training exercises. However, the extent to which metabolomic responses are shared across those diverse training exercises is unknown.

Environments and stressors vary across military training exercises, and service members are often required to subsist on rations designed for general use irrespective of mission type. Understanding common metabolic responses could inform the development of nutrition strategies and combat rations tailored toward better-supporting resilience to and recovery from common physiological demands experienced across different military training and operational environments (5). The objective of this study was to integrate metabolomic data collected from three studies conducted during distinct real or simulated military training exercises to identify shared metabolomic signatures. In addition, we investigated the relationships between shared metabolomic signatures and traditional biomarkers of inflammation, tissue damage, and anabolic status.

## materials and methods

### Experimental Designs and Study Participants

**Figure 1.**
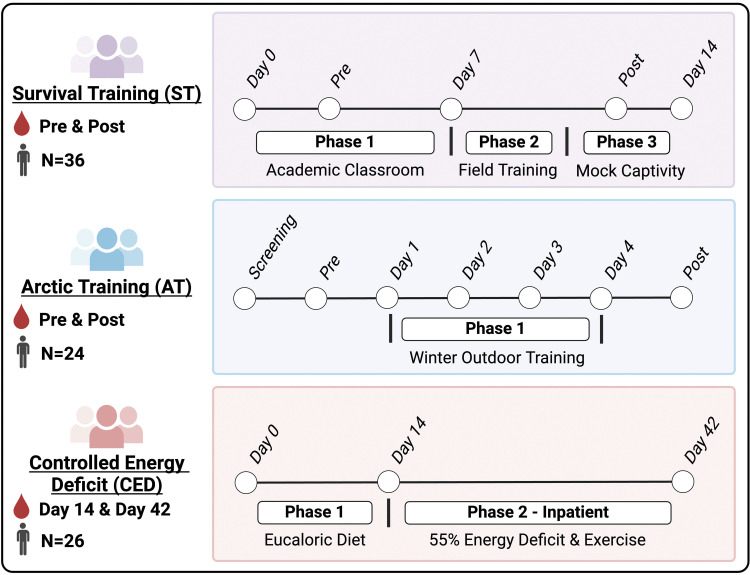
Timelines for ST, AT, and CED studies included in the comparative analysis. Figure was created with BioRender.com.

This study was a secondary analysis of individual participant data derived from metabolomic datasets generated during three previous studies conducted by the United States Army Research Institute of Environmental Medicine (USARIEM; Natick, MA) Military Nutrition Division and Pennington Biomedical Research Center (Baton Rouge, LA). Study data were reanalyzed using a common data analysis protocol to enable comparisons across studies. The overall approach is shown in the Graphical Abstract. Individual study timelines are represented in Fig. 1. Written informed consent was obtained for all study participants, as described in detail elsewhere (12–14). Brief study descriptions are included herein and more detailed descriptions are included within the primary publications cited for each study.

The survival training (ST) dataset was from a randomized, double-blind, placebo-controlled study that assessed the effects of tyrosine supplementation on stress responses in individuals undergoing survival, evasion, resistance, and escape (SERE) (12). Briefly, the training lasted 2 wk and was separated into three distinct phases: a week-long educational component in a classroom setting, several days of survival field training in a natural environment, and several days of captivity simulating a prisoner of war scenario. During the classroom portion, students received training on survival, evasion, resistance, and escape techniques. Physical training and diet were at the student’s discretion. During the field training, students first completed a survival exercise during which energy intake and sleep were limited and physical activity was high. Students were then “captured” and spent several days within the captivity scenario. During captivity, students applied their learned skills in an environment simulating that which might be experienced as a prisoner of war. Energy intake and sleep were severely restricted, and students experienced extreme physiological and psychological stress (12). Although not measured in the study, studies conducted in other SERE training environments have estimated energy deficits of ∼4,000 kcal/day resulting from energy restriction and high energy demands (15). For this project, data derived from 36 male individuals in the placebo group were included (Table 1). Pretraining samples were obtained during the classroom phase of training, and posttraining samples were obtained after the completion of two mock interrogations during the captivity phase. The pretraining samples were collected under fasting conditions, whereas the posttraining samples were collected during a period when food intake was severely restricted. The study protocol was approved by institutional review boards at both the National Naval Medical Center and USARIEM. The trial was registered through clinicaltrials.gov (NCT01913925). Primary and secondary study outcomes have been reported elsewhere (12).

**Table 1.** Participant demographics

	Survival Training	Arctic Training	Controlled Energy Deficit
Participants, *n*	36	24	26
Age, yr	25 ± 5	19 ± 1	25 ± 5
Height, cm	179 ± 6	183 ± 5	178 ± 6
Baseline body weight, kg	83.7 ± 6.5	78.4 ± 7.4	76.3 ± 10.2
Baseline BMI, kg/m^2^	26.1 ± 1.6	23.3 ± 1.8	24.0 ± 3.0
Change in body weight, kg	−6.2 ± 1.3	−2.7 ± 1.0	−5.8 ± 1.3

Data are means ± SD. Demographic information for survival training, artic training, and controlled energy deficit studies. BMI, body mass index.

The arctic training (AT) dataset was from a randomized, double-blind, placebo-controlled study that investigated whether providing supplemental nutrition products varying in macronutrient composition would differentially attenuate whole body protein balance and correct negative energy balance during a 4-day, 51-km cross-country ski march (14). Air temperatures during the ski march ranged from −17°C to −3°C (16). Sleep duration in a subset of participants was estimated to be 5–6 h/night (16). Energy expenditures in this cohort averaged 6,080 kcal/day, resulting in a 3,250 kcal/day energy deficit (17). For this analysis, study groups were combined as all three nutritional interventions tested elicited similar metabolomic responses, as detailed elsewhere (9), resulting in a sample size of 24 (Table 1). Pretraining and posttraining blood samples were obtained under fasting conditions before and immediately after the training exercise. The study protocol was approved by the institutional review boards at USARIEM and the Regional Committees for Medical and Health Research Ethics (REK, sØr-Øst, Oslo, Norway). The trial was registered through clinicaltrials.gov (NCT0232708). Primary and secondary study outcomes have been reported elsewhere (9, 14, 16–18).

The controlled energy deficit (CED) dataset was derived from a randomized, double-blind, placebo-controlled trial that assessed the effects of testosterone supplementation on exercise- and diet restriction-induced alterations in body composition and muscle function (13, 19). Briefly, this 56-day experiment comprised three phases: a 2-wk long eucaloric, controlled-feeding run-in phase, a 4-wk 55% energy deficit residential phase, and a 2-wk long recovery phase. The energy deficit phase was designed to reproduce the severe energy deficits observed during military training exercises. Accordingly, exercise-induced energy expenditure was increased by 50% from that measured during the run-in phase using a combination of low-, moderate-, and high-intensity aerobic exercise (40–85% V̇o_2peak_). The exercise was performed in 1–4 prescribed sessions daily that included a variety of modalities, including walking with a weighted vest, running, cycle ergometry, and elliptical training. Diets were then prescribed and provided to achieve the desired energy deficit (13, 19). Testing was conducted under normal ambient temperatures, and sleep was unrestricted. Energy expenditures during the energy deficit phase averaged 3,682 kcal/day, resulting in a 1,935 kcal/day energy deficit (19). For this analysis, the placebo group (*n* = 26) that received weekly injections of a placebo (sesame oil) instead of testosterone enanthate was included (Table 1). Fasting blood samples were obtained at the beginning and end of the energy deficit phase. The study protocol was approved by the institutional review boards at USARIEM and the Pennington Biomedical Research Center. The trial was registered through clinicaltrials.gov (NCT02734238). Primary and secondary study outcomes have been reported elsewhere (8, 19–27).

### Circulating Metabolomics and Biomarkers

Blood samples were processed into plasma (ST and AT studies) or serum (CED study) and immediately frozen at −80°C. Frozen samples were later shipped overnight on dry ice to Metabolon, Inc. (Durham, NC) for untargeted metabolomic profiling, which has been previously described in detail (6, 17). Briefly, both reverse-phase and hydrophilic interaction chromatography ultrahigh-performance liquid chromatography-tandem mass spectrometry (RP/UPLC-MS/MS and HLIC/UPLC-MS/MS) with positive and negative electrospray ionization modes were performed using a Waters ACQUITY UPLC and a Thermo Scientific Q-Exactive Orbitrap LC-MS/MS. To ensure adequate quality control, replicates, internal standards, and blanks were used. Peaks were extracted from raw data and identified according to retention time, mass-to-charge ratio (m/z), and MS/MS forward and reverse scores when compared to purified standards maintained through Metabolon’s proprietary library. After metabolites were identified, peak areas were determined and used for all downstream analyses.

Circulating biomarkers, including C-reactive protein (CRP), ferritin, hepcidin, sex hormone-binding globulin (SHBG), testosterone, free testosterone, insulin-like growth factor 1 (IGF-1), lactate dehydrogenase (LDH), myoglobin, creatine kinase (CK), and epinephrine, were measured using chemiluminescent immunometric assays, enzyme-linked immunosorbent assays, or automated chemistry analyzers as previously reported (17–19). These biomarkers were selected for this analysis based on available data and their representation of physiological responses known to be induced during military training exercises that include inflammation, muscle damage, and suppression of the hypothalamic–pituitary–gonadal axis. Not all biomarkers were measured in each dataset. All metabolomic and blood biomarker data were generated previously during the parent protocols, and the existing datasets were used to meet the objectives of this analysis.

### Statistical Analyses

Sample size calculations were conducted for primary study outcomes, which have been previously described for each respective study (12–14, 17). Statistical analyses were conducted and figures were generated using R v 4.1.2 and RStudio v 2021.09.2 (R Core Team), GraphPad Prism 9.4.0, MetaboAnalyst 5.0 (28), Venny 2.1 (Oliveros, J.C.), and BioRender. For each study, circulating biomarker data were log-transformed and analyzed using paired *t* tests. Metabolomics data from each study were processed separately using the same protocol (Graphical Abstract). Within each dataset, data were median-scaled to adjust for batch effects, followed by the removal of metabolites with >25% missing values, imputed with 1/5 the minimum positive value for any remaining missing values, filtered, log_10_ transformed, and auto-scaled. Paired two-sample *t* tests with a false discovery rate (FDR) of 0.05 were then carried out within each study to determine which metabolites were significantly altered during each training. Fold change metrics based on log_2_ values were then calculated and used to construct volcano plots. Principal component analysis was used to assess global changes in metabolomic profiles. To determine which families of metabolites were most impacted by each training regimen, significantly altered metabolites were grouped together based on superpathways and subpathways as determined by Metabolon, Inc. The top 50 largest positive and negative significant fold changes were then extracted (Supplemental Figs. S1 and S2), followed by comparisons of all significantly altered metabolites across the studies using Venn diagrams. Repeated measure correlations were conducted to assess associations between metabolites and biomarkers (29, 30). Data are presented as min-max floating bar plots with lines at the median or as the mean (SD). All data were FDR-corrected with an adjusted *P* value set at 0.05, except the plasma biomarker data, where significance was determined by a *P* value <0.05.

## RESULTS

### Plasma Biomarkers

**Figure 2.**
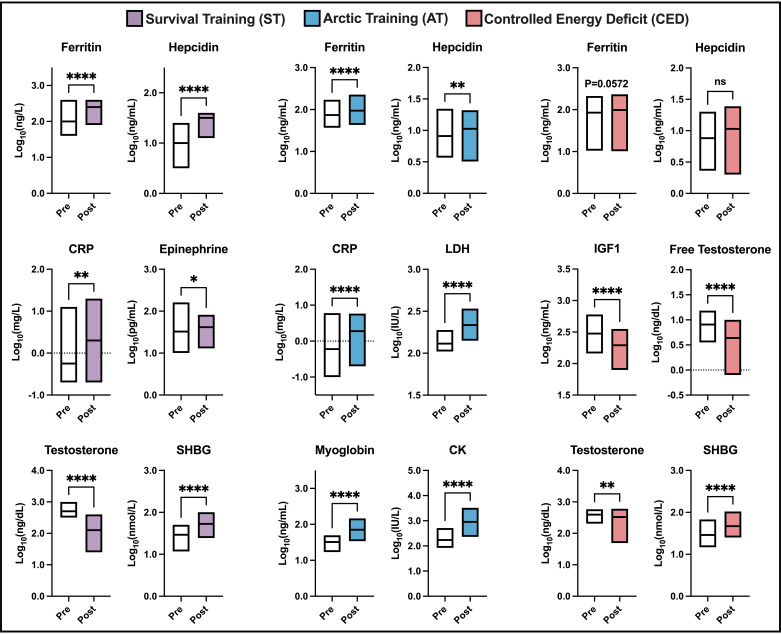
Military training induced alterations in biomarkers of inflammation, stress, tissue damage, and anabolic hormones. Not all biomarkers were measured in all studies, and the results shown reflect whether a biomarker was measured in that particular study. Values are presented as medians (min-max); ST: *n* = 36, AT: *n* = 24, and CED: *n* = 26. Data were analyzed by a paired *t* test. *P < 0.05, **P < 0.01, ****P < 0.0001. CRP, C-reactive protein; CK, creatine kinase; IGF-1, insulin-like growth factor 1; LDH, lactate dehydrogenase; SHBG, sex hormone-binding globulin.

Biomarkers related to inflammation, stress, and tissue damage significantly increased in response to ST and AT (*P* < 0.05) (Fig. 2). C-reactive protein (CRP), ferritin, and hepcidin increased in ST and AT (*P* < 0.05), whereas ferritin trended toward an increase in CED (*P* = 0.06). Epinephrine (*P* < 0.05) increased in ST, whereas lactate dehydrogenase, creatine kinase (CK), and myoglobin (measures of muscle damage) increased in AT (*P* < 0.0001). Testosterone concentrations were decreased at the end of ST and CED, whereas sex hormone-binding globulin concentrations were increased (*P* < 0.05).

### Global Plasma Metabolomic Profiles

**Figure 3.**
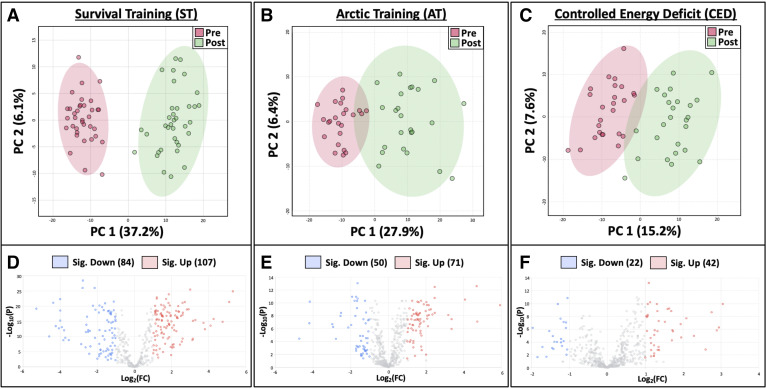
Overview of metabolomic profiles for each study using principal component analysis (PCA) plots (*A−C*) and volcano plots (*D−F*). Red indicates pretraining samples; green indicates posttraining samples. For volcano plots, a FDR of 0.05 and a fold change (FC) threshold of 1 or −1 were used for all studies. All FC analyses were relative to the pretraining time point and represented log_2_FC values. Blue indicates a decreased log_2_FC; red indicates an increased log_2_FC. *A* and *D*: survival training (ST; *n* = 36). *B* and *E*: artic training (AT; *n* = 24). *C* and *F*: controlled energy deficit (CED; *n* = 26). Figure was created with BioRender.com. FDR, false discovery rate.

**Figure 4.**
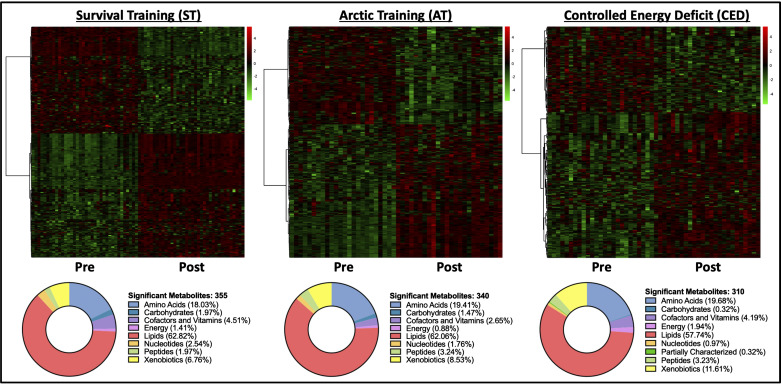
Heatmaps of each study with donut charts comprising all significantly altered metabolites categorized by superpathway. Paired *t* tests with a FDR of 0.05 were used to determine significance. Red indicates that a metabolite is increased; green indicates that a metabolite is decreased. ST: *n* = 36, AT: *n* = 24, and CED: *n* = 26. Figure was created with BioRender.com. FDR, false discovery rate.

The total number of identified metabolites in the ST, AT, and CED datasets was 687, 737, and 958, respectively. Global shifts in circulating metabolomic profiles were observed in all three studies (Fig. 3, *A*–*C*). Volcano plots (Fig. 3, *D*–*F*) revealed significant log_2_ fold changes of >1 or <−1 for 191 metabolites in ST, 121 metabolites in AT, and 64 metabolites in CED. Of the >300 metabolites significantly altered following training within each study, most were part of lipid (57–63% of significant metabolites) and amino acid (18–20% of significant metabolites) superpathways (Fig. 4 and Supplemental Tables S1, S2, and S3).

### Metabolomic Pathway Analysis

**Figure 5.**
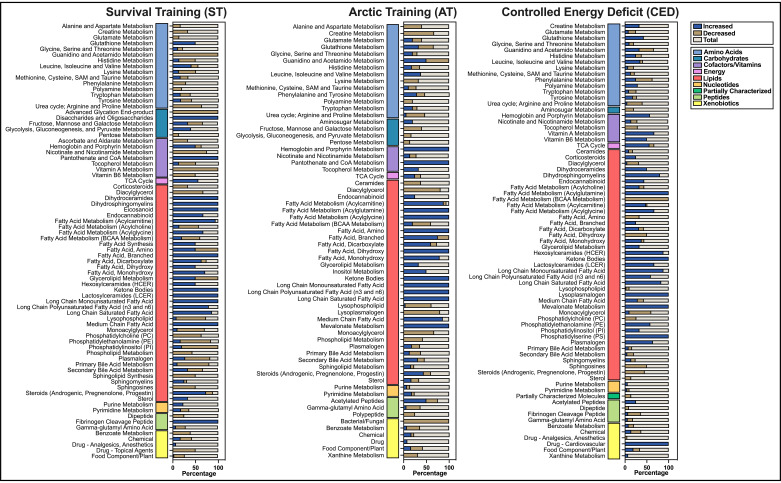
Subpathway enrichment alterations demonstrated shared increases in fatty acids, ketones, and acylcarnitines among the studies. The subpathways are organized by superpathway as shown in the legend. For all plots, paired tests with a FDR of 0.05 was used. FDR, false discovery rate; TCA, tricarboxylic acid.

Metabolites related to the ketone body, acylcarnitine, acylglycine, and medium- and long-chain fatty acid metabolism subpathways were among those that consistently increased across all three exercises (Fig. 5). By contrast, metabolites related to mono- and di-acylglycerol and various phospholipid subpathways were among those that consistently decreased across all three exercises (Fig. 5).

**Figure 6.**
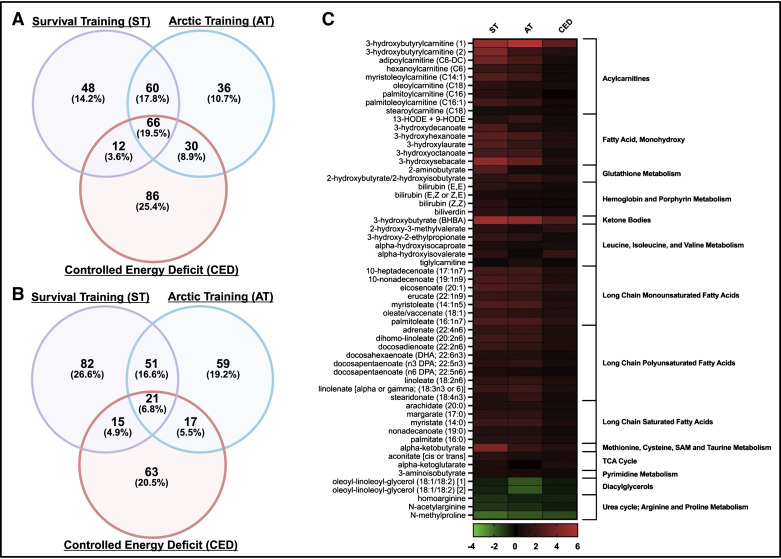
*A* and *B*: Venn diagrams showing shared significantly increased (*A*) and decreased (*B*) metabolites across the studies. *C*: log_2_FC values of representative shared metabolites. All FC analyses were relative to the pretraining time point. For all plots, paired samples *t* tests with an FDR of 0.05 were used. Figure was created with BioRender.com. FC, fold change; FDR, false discovery rate.

Changes in 87 metabolites were shared across studies. Within this panel of shared metabolites, 66 (20%) increased (Fig. 6*A* and Supplemental Table S4) and 21 (7%) decreased (Fig. 6*B* and Supplemental Table S5) across all exercises. The largest increases in shared metabolites were observed within the acylcarnitine subpathway, with 3-hydroxybutyrylcarnitine (1) and (2) demonstrating some of the highest log_2_ fold changes, followed by adipoylcarnitine (Supplemental Table S6). Similarly, the ketone body, 3-hydroxybutyrate (BHBA), demonstrated the largest increases in response to all training regimens (log_2_ fold change: ST: 5.30, AT: 4.69, and CED: 2.75) (Fig. 6*C* and Supplemental Fig. S1). Compounds within major lipid groups, including long-chain fatty acids and monohydroxy fatty acids, were also among the shared metabolites found to increase in all three datasets (Fig. 6*C* and Supplemental Fig. S1). Among the polyunsaturated fatty acids (PUFAs), increases in linoleate, linolenate, and various related downstream fatty acids were consistently observed. Metabolites related to the metabolism of the antioxidant glutathione, such as 2-aminobutyrate and 2-hydroxy(iso)butyrate, were also increased in all three studies, as were the metabolites 13-hydroxyoctadecadienoic acid (13-HODE) + 9-hydroxyoctadecadienoic acid (9-HODE), which have been linked to oxidative stress and inflammation (31, 32). Other noteworthy changes in the shared metabolites included increases in hemoglobin and porphyrin metabolism products, branched-chain amino acid (BCAA) metabolites, α-ketobutyrate, and 3-aminoisobutyrate (Fig. 6*C*, Supplemental Fig. S1, and Supplemental Table S4).

### Associations Among Shared Metabolites and Biomarkers

**Figure 7.**
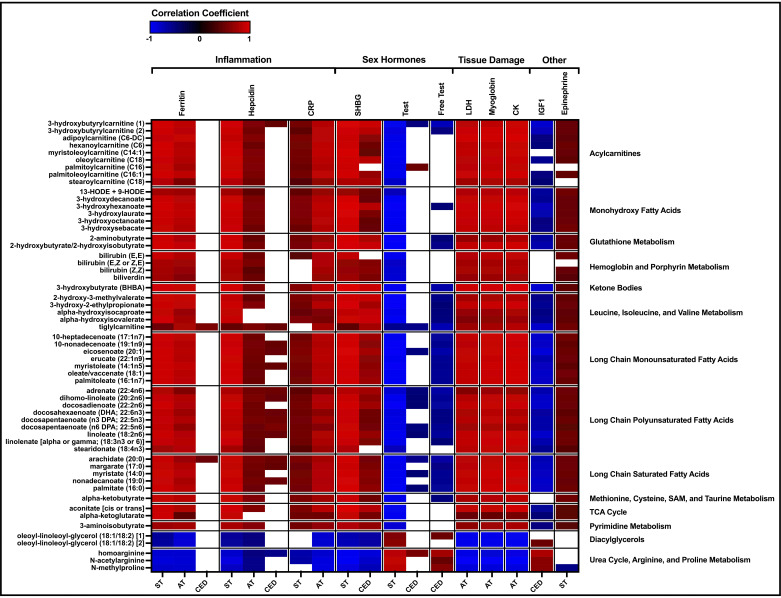
Representative shared metabolites significantly correlated with biomarkers of inflammation, tissue damage, stress, and anabolic hormones. Not all biomarkers were measured in all studies, and the results shown reflect whether a biomarker was measured in that particular study. White spaces indicate a FDR of ≥0.05. Values are repeated-measures correlation coefficients. ST: *n* = 36, AT: *n* = 24, and CED: *n* = 26. CRP, C-reactive protein; FDR, false discovery rate; IGF-1, insulin-like growth factor 1; LDH, lactate dehydrogenase; SHBG, sex hormone-binding globulin.

Correlation analyses explored associations between the shared metabolites and changes in biomarkers of inflammation, tissue damage, and anabolic hormones and the persistence of those relationships across studies (Fig. 7). Correlation patterns revealed positive associations among many fatty acid-associated metabolites with markers of inflammation and tissue damage. Many of the same metabolites demonstrated inverse associations with anabolic hormones (Supplemental Tables S7–S9). Similar patterns were observed for multiple BCAA and glutathione metabolites (Fig. 7).

## DISCUSSION

The primary findings of this study were that the circulating metabolome was noticeably altered in all three training scenarios, and a panel of 87 metabolites was shared across the studies. Within that panel, the largest magnitude changes were observed for metabolites related to fatty acid metabolism, acylcarnitine metabolism, ketone metabolism, and oxidative stress. Many of those metabolites positively correlated with established biomarkers of inflammation and tissue damage and inversely with anabolic hormones. Collectively, these findings provide initial insight into a potential common metabolic signature of the physiological demands resulting from military training. That signature is indicative of increased fatty acid metabolism and oxidative stress and is associated with inflammation, tissue damage, and suppression of anabolic signaling.

Although widespread metabolic responses were detected in each study, changes in metabolites related to lipid metabolism predominated. These changes were primarily characterized by increases in metabolites within pathways of acylcarnitine, ketone, monohydroxy fatty acid, medium-chain fatty acid, and long-chain fatty acid metabolism. Findings are similar to those reported following single endurance exercise bouts (33–36), multisession exercise training (37), and during fasting (38), and are suggestive of increased lipolysis and fatty acid oxidation from adipose depots, skeletal muscle, and the liver (39). Increased fatty acid oxidation results in the hepatic accumulation of acetyl-CoA and subsequent generation of ketone bodies, such as acetoacetate and BHBA, which may then be converted into 3-hydroxybutyrylcarnitine (40). Accordingly, BHBA and 3-hydroxybutyrylcarnitine were among the shared metabolites, demonstrating the largest fold changes within each study. We previously reported that increases in the same metabolites were correlated with the magnitude of energy deficit and body mass loss, but not energy expenditure or energy intake within the AT dataset (9). The observation that fold changes in these metabolites were numerically larger in the ST and AT datasets compared with the CED dataset may therefore reflect differences in the magnitude of energy deficit, though other factors such as differences in plasma versus serum compartmental composition, ambient environment, and sleep deprivation cannot be ruled out (41, 42).

In addition to 3-hydroxybutyrylcarnitine, large increases in several acylcarnitines were observed, particularly in the ST and AT datasets. Increases in many of these acylcarnitine species have been found in other exercise-related studies (36, 43, 44) and may be released into circulation in a tissue-specific manner (45). Elevated concentrations of acylcarnitine metabolites in response to exercise stress are indicative of increased fatty acid oxidation, as their primary function is to facilitate the transport of fatty acids across mitochondrial membranes during β-oxidation (46). However, a large overlap in BCAA and acylcarnitine metabolites exists, and increased concentrations of acylcarnitines have also been associated with a metabolic shift from an anabolic to a catabolic state (47). Indeed, our laboratory has recently reported concurrent increases in BCAA and acylcarnitine metabolites when aerobic exercise was initiated in volunteers with low glycogen availability (10). We hypothesized that those findings may indicate a greater reliance on BCAAs for oxidative purposes to sustain physical performance when endogenous carbohydrate stores are low. Beyond being a marker for catabolism, studies have also linked increased acylcarnitine metabolite concentrations to inflammation and insulin resistance (48, 49). For example, a dose-dependent increase in IL-6 production, with a concurrent rise in markers of cell permeability and death of differentiated C_2_C_12_ myotubes, was reported in one study using an in vitro model where long-chain acylcarnitines were enriched in cell culture media (50). Collectively, these data suggest that although increased acylcarnitine metabolite concentrations during strenuous military training exercises under energy deficit conditions are a normal metabolic response to facilitate greater fatty acid oxidation, a rise in these metabolites may also signify increased protein catabolism and increased systemic inflammation (51). These results provide additional evidence (14, 52) supporting the need to mitigate energy deficit severity during military training exercises.

The shared metabolic responses observed within pathways of polyunsaturated fatty acid (PUFA) mobilization, oxidized linoleic acid metabolite (OXLAM) formation, and increased glutathione metabolism likely reflect intersecting biochemical pathways related to oxidative stress. In response to numerous stimuli, including exercise, phospholipases remodel lipid membranes, liberating unsaturated fatty acids such as linoleic acid (C18:2n6) and α-linolenic acid (C18:3n3) (53). Through a series of interconversions mediated by lipoxygenases (LOX), linoleic acid can be converted into 9-hydroxyoctadecadienoic acid (9-HODE) and 13-hydroxyoctadecadienoic acid (13-HODE), which serve multifunctional roles as peroxisome-proliferator-activated receptor gamma (PPAR-γ) agonists and are indicators of oxidative stress in response to exercise and a variety of diseases (54, 55). Herein, modest increases in circulating linoleic acid and 13-HODE + 9-HODE were observed in all studies, along with increases in 2-hydroxybutyrate, 2-aminobutyrate, and α-ketobutyrate. The latter three compounds are key intermediates generated from the transsulfuration pathway during glutathione synthesis in response to oxidative stress (56–58). Collectively, these results indicate increased activation of oxidative stress-related pathways during all of the exercises. Furthermore, these metabolites correlated with markers of inflammation and tissue damage, mainly in the AT and ST datasets. In the CED dataset, many of these metabolites were inversely associated with changes in circulating anabolic hormones. Excessive oxidative stress can reduce muscle force generation under high-intensity conditions and impair recovery (59, 60). Thus, altering oxylipin generation or improving glutathione recycling may be potential intervention targets for reducing oxidative stress during or following arduous military training. Potential interventions could include dietary supplements such as *N*-acetyl-cysteine and fish oil or modulating the fatty acid composition of the diet. Indeed, recent systematic reviews have concluded that although evidence is currently limited and not entirely consistent, *N*-acetyl-cysteine supplementation has shown potential for having antioxidant effects, regulating glutathione homeostasis, and improving physical performance in healthy men (61, 62), whereas omega-3 supplementation may improve recovery from physiological stress in healthy adults (63, 64). Whether such interventions meaningfully impact physical performance and recovery outcomes in military populations warrants consideration for future research (65).

This analysis is, to our knowledge, the first comparative analysis of metabolomic responses across distinct military training scenarios. Strengths include the use of individual participant data, the same metabolomics platform, and a common analytical approach across datasets. However, the findings should be interpreted within the context of several limitations. First, due to updates in databases over time, not all metabolites were included in all datasets, and changes in nomenclature may have caused some shared metabolites to be overlooked. Similarly, not all biomarkers were measured in all three studies. In addition, two datasets used plasma, whereas one study used serum, preventing quantitative comparisons between datasets. Collectively, this prevented a complete examination of shared metabolites and correlations across datasets. Furthermore, although the analytical approach was designed to identify the metabolites sharing the strongest signals across studies, the untargeted analysis, moderate power, conservative FDR threshold, and innate variability related to the study environments collectively reduced the common metabolic signatures detected. This issue was likely compounded by interindividual and intraindividual variability in metabolic responses attributable to uncontrolled environmental factors in the AT and ST datasets. Notably, however, shared metabolic signatures were detectable despite that variability, suggesting that the signals may be reproducible across disparate military training exercises. Lastly, the cohorts of all three studies were exclusively comprised of men, which reduces generalizability given that sex differences in metabolomic responses to military training have recently been reported (66).

Using untargeted metabolomic profiling to assess metabolic responses to physical activity, diet interventions, and other behavioral or environmental exposures in both the general population and military cohorts is increasingly common. The results of these efforts are contributing to an emergent understanding of how the complex, highly contextual metabolome relates to biological processes affected by exercise, diet, and the environment. This comparative metabolomics analysis of three distinct real and simulated military training exercises suggests a potential common metabolic signature of arduous military training characterized primarily by increased fatty acid oxidation and oxidative stress. Study findings demonstrate that these metabolomic signatures may be consistent and reproducible despite key differences in study design and environment while implicating energy deficit as a driving factor with other stressors such as physical and psychological demands, limited sleep, and environmental stress as possible contributors. Validation of these findings within other military training exercises, including those with female cohorts, is warranted to definitively identify common metabolomic signatures reflecting the unique physiological demands of military training.

## DATA AVAILABILITY

The data that support the findings of this study are available from the corresponding author upon reasonable request pending legal and ethical approvals.

## SUPPLEMENTAL DATA

10.6084/m9.figshare.25473895 Supplemental Tables S1–S9 and Supplemental Figs. S1 and S2: https://doi.org/10.6084/m9.figshare.25473895.

## GRANTS

This work was funded by the United States Army Medical Research and Development Command and the Collaborative Research to Optimize Warfighter Nutrition II and III Projects.

## DISCLAIMERS

The opinions or assertions contained herein are the private views of the authors and are not to be construed as official or reflecting the views of the United States (US) Army or the Department of Defense. Any citations of commercial organizations and trade names in this report do not constitute an official Department of the Army endorsement or approval of the products or services of these organizations. This article was prepared while S.M.P. was employed at the US Army Research Institute of Environmental Medicine. The opinions expressed in this article are the author’s own and do not reflect the view of the National Institutes of Health, the Department of Health and Human Services, or the US Government. M.D. was funded by an appointment to the US Army Research Institute of Environmental Medicine administered by the Oak Ridge Institute for Science and Education through an interagency agreement between the US Department of Energy and the US Army Medical Research and Development Command.

## DISCLOSURES

No conflicts of interest, financial or otherwise, are declared by the authors.

## AUTHOR CONTRIBUTIONS

M.D., L.M.M., J.C.R., H.R.L., S.M.P., and J.P.K. conceived and designed research; M.D., L.M.M., J.C.R., H.R.L., S.M.P., and J.P.K. performed experiments; M.D. and J.P.K. analyzed data; M.D., L.M.M., and J.P.K. interpreted results of experiments; M.D. prepared figures; M.D., L.M.M., and J.P.K. drafted manuscript; M.D., L.M.M., J.C.R., H.R.L., S.M.P., and J.P.K. edited and revised manuscript; M.D., L.M.M., J.C.R., H.R.L., S.M.P., and J.P.K. approved final version of manuscript.
